# Predicting the survival probability of functional neuroendocrine tumors treated with peptide receptor radionuclide therapy: Serbian experience

**DOI:** 10.3389/fendo.2023.1270421

**Published:** 2024-01-04

**Authors:** Vladimir Vukomanovic, Katarina Vuleta Nedic, Marija Zivkovic Radojevic, Aleksandar Dagovic, Neda Milosavljevic, Marina Markovic, Vladimir Ignjatovic, Ivana Simic Vukomanovic, Svetlana Djukic, Marijana Sreckovic, Milena Backovic, Marko Vuleta, Aleksandar Djukic, Verica Vukicevic, Vesna Ignjatovic

**Affiliations:** ^1^ Department of Nuclear Medicine and Clinical Oncology, Faculty of Medical Sciences, University of Kragujevac, Kragujevac, Serbia; ^2^ Department for Nuclear Medicine, University Clinical Center Kragujevac, Kragujevac, Serbia; ^3^ Department for Radiotherapy, University Clinical Center Kragujevac, Kragujevac, Serbia; ^4^ Department for Medical Oncology, University Clinical Center Kragujevac, Kragujevac, Serbia; ^5^ Department of Internal Medicine, Faculty of Medical Sciences, University of Kragujevac, Kragujevac, Serbia; ^6^ Clinic for Cardiology, University Clinical Center Kragujevac, Kragujevac, Serbia; ^7^ Department of Social Medicine, Faculty of Medical Sciences, University of Kragujevac, Kragujevac, Serbia; ^8^ Department of Health Promotion, Institute of Public Health, Kragujevac, Serbia; ^9^ Clinic for Hematology, University Clinical Center Kragujevac, Kragujevac, Serbia; ^10^ Department of Medical and Business-Technological, Academy of Professional Studies Sabac, Sabac, Serbia; ^11^ Department for Pathology, Faculty of Medicine, University of Belgrade, Belgrade, Serbia; ^12^ Department for Cardiology, Clinical Hospital Center “Dr Dragisa Misovic Dedinje”, Belgrade, Serbia; ^13^ Department of Pathophysiology, Faculty of Medical Sciences, University of Kragujevac, Kragujevac, Serbia; ^14^ Clinic for Endocrinology, Diabetes and Metabolic Diseases, University Clinical Center Kragujevac, Kragujevac, Serbia; ^15^ Emergency Medical Institute, Belgrade, Serbia

**Keywords:** NET, PRRT, functional tumors, overall survival, progression free survival

## Abstract

**Introduction:**

Peptide receptor radionuclide therapy (PRRT) is a treatment option for well-differentiated, somatostatin receptor positive, unresectable or/and metastatic neuroendocrine tumors (NETs). Although high disease control rates seen with PRRT a significant number NET patients have a short progression-free interval, and currently, there is a deficiency of effective biomarkers to pre-identify these patients. This study is aimed at determining the prognostic significance of biomarkers on survival of patients with NETs in initial PRRT treatment.

**Methodology:**

We retrospectively analyzed 51 patients with NETs treated with PRRT at the Department for nuclear medicine, University Clinical Center Kragujevac, Serbia, with a five-year follow-up. Eligible patients with confirmed inoperable NETs, were retrospectively evaluated hematological, blood-based inflammatory markers, biochemical markers and clinical characteristics on disease progression. In accordance with the progression og the disease, the patients were divided into two groups: progression group (n=18) and a non-progression group (n=33). Clinical data were compared between the two groups.

**Results:**

A total of 51 patients (Md=60, age 25-75 years) were treated with PRRT, of whom 29 (56.86%) demonstrated stable disease, 4 (7.84%) demonstrated a partial response, and 14 (27.46%) demonstrated progressive disease and death was recorded in 4 (7.84%) patients. The mean PFS was a 36.22 months (95% CI 30.14-42.29) and the mean OS was 44.68 months (95% CI 37.40-51.97). Univariate logistic regression analysis displayed that age (p<0.05), functional tumors (p<0.05), absolute neutrophil count (p<0.05), neutrophil-lymphocyte ratio-NLR (p<0.05), C-reactive protein-CRP (p<0.05), CRP/Albumin (p<0.05), alanine aminotransferase-ALT (p<0.05), were risk factors for disease progression. Multivariate logistic regression analysis exhibited that functional tumors (p<0.001), age (p<0.05), CRP (p<0.05), and ALT (p<0.05), were independent risk factors for the disease progression in patients with NETs. Tumor functionality was the most powerful prognostic factor. The median PFS (11.86 ± 1.41 vs. 43.38 ± 3.16 months; p=0.001) and OS (21.81 ± 2.70 vs 53.86 ± 3.70, p=0.001) were significantly shorter in patients with functional than non-functional NETs respectively.

**Conclusion:**

The study’s results suggest that tumor functionality, and certain biomarkers may serve as prognostic survival indicators for patients with NETs undergoing PRRT. The findings can potentially help to identify patients who are at higher risk of disease progression and tailor treatment strategies accordingly.

## 1 Introduction

Neuroendocrine tumors (NETs) are a heterogenous group of tumors originating from widely distributed neuroendocrine cells that have both “neuro” and “endocrine” features (1). This entity with a broad spectrum of clinical manifestations and complex histopathological characteristics differs in grade, differentiation, functional status, and primary site (2). Although the biological behavior of the majority of well-differentiated NETs is relatively indolent, others may be more aggressive and associated with poor prognosis (3). Over the past few decades, peptide receptor radionuclide therapy (PRRT) with radiolabeled somatostatin analogs (SSAs) has gain momentum in the management of inoperable or metastatic, well-differentiated NETs that express somatostatin receptors (SSTR). The range of indication for PRRT was expanded overtime, from gastroenteropancreatic (GEP) NETs to the treatment of SSR positive bronchopulmonary NETs (BP-NETs), paraganglioma and medullary thyroid cancers (4–6). It was shown in NETTER-1 trial that PRRT plus long-acting octreotide improve progression-free survival (PFS) and overall survival (OS) in advanced midgut NETs in comparison to high dose of long-acting octreotide treatment alone (7). Despite high disease control rates seen with PRRT, a subset of the NET population will not respond to radionuclide therapy or even disease progression will be registered (8). Therefore, in order to predict the anti-tumor effect of PRRT, it is necessary to determine reliable response predictors including clinical parameters, biomarkers or imaging (9). Recently, it has been more obvious that inflammatory response also affects tumor growth and patient outcomes (10). Several studies have pointed out the prognostic role of hematological and other blood-based markers of inflammation, including neutrophil/lymphocyte ratio (NLR), platelet/lymphocyte ratio (PLR), CRP/albumin ratio (CRP/Alb) in treatment outcomes of patients receiving 177-Lu based PRRT (9–11). The objective of this study is to evaluate the prognostic abilities of inflammatory and other clinical markers in patients with neuroendocrine tumors who are initiating PRRT.

## 2 Materials and methods

This retrospective study included 51 NET patients who received PRRT in the University Clinical Center Kragujevac, Serbia, covering a 5-year follow-up period (2018-2023). All patients were evaluated and determined to be eligible for PRRT by a dedicated NETs Tumor Board at the University Clinical Center Serbia, Belgrade. The main inclusion criteria were pathologically and clinically confirmed NET with positive SSTR-based imaging (^99m^Tc-HYNIC-TOC). Patients with autoimmune diseases and other primary tumors were excluded. The Ethics Committee of the University Clinical Center Kragujevac approved the study (01/23-132).

Patients data as age, gender, tumor localization, pathological findings (WHO classification, tumor size, lymph node metastasis, histological grade, and mitosis), and distant metastasis were collected from the electronic medical records system. Peripheral blood tests (blood count, liver and kidney function, albumin level, CRP, hormonal secretion) were performed before first PRRT. Based on their origin, NETs were categorized into three groups: GEP, lungs, and other organs. Tumor grade was classified as grade 1, grade 2, or grade 3 (12). Tumor functionality was assessed based on the presence of typical clinical symptoms associated with carcinoid syndrome (facial flushing, abdominal pain, diarrhea, bronchospasm) and elevated 24-hour urine levels of 5-hydroxyindoleacetic acid (5-HIAA), chromogranin (CgA) and NSE (13).

There was an interval of 4-6 weeks between the use of long-acting SSA and PRRT. PRRT was administered following a standardized Lu-177 based protocol with a dosage of 5.55 GBq per cycle. The cycles were repeated at intervals of 8-12 weeks, mostly DOTA-octreotate based SSA with median cumulative activity of 22 GBq, median four cycles. Renal protection with an amino acid-based solution was administered during the PRRT treatment.

### 2.1 Determination of biochemical and hematological parameters

The concentrations of biochemical markers were measured using standard methods in Laboratory diagnostic service of the University Clinical Center Kragujevac. Serum concentrations of ferritin, C-reactive protein-CRP, aspartate aminotransferase-AST, alanine aminotransferase-ALT, creatine kinase-CK, lactate dehydrogenase-LDH, renal function test (urea and creatinine) were determined by the reagents (Beckman Coulter Inc. Brea, USA) certified and validated for the use on Olympus AU680 Analyzer. Using the blood count results: platelets and absolute counts of white blood cells subtypes (neutrophils, monocytes, lymphocytes), the indices were computed: platelet-to-lymphocyte ratio (PLR), neutrophil-to-lymphocyte ratio (NLR), and systemic inflammation response index (SIRI). SIRI was defined as multiplication of neutrophils and monocytes divided by lymphocytes count. We assessed CRP to albumin ratio (CRP/Alb) by dividing CRP in mg/L through albumin in g/L. Plasma chromogranin A (CgA) was assessed by ELISA kit, serum neuron-specific enolase-NSE was measured by an immunoradiometric assay (IRMA), 5-hydroxy-3-indoleacetic acid-5-HIAA was measured in 24-hour excreted urine by ELISA.

### 2.2 Follow-up

Evaluation of response to therapy was done using contrast-enhanced MDCT or MRI (4-8 weeks after 2 applied cycles). The results of PRRT were interpreted according to RECIST 1.1. According to the response to the therapy, the patients were divided into two groups, the group with progression (PD) and the group without progression (SD or PR). The primary endpoints were overall survival (OS) and Progression free survival (PFS). OS was defined as the interval between the date of first PRRT and death from any cause. PFS was defined as the time from the date of the first-PRRT to the time of the disease progression.

### 2.3 Statistics

The collected data underwent descriptive statistical analysis methods. Significance of difference for continuous variables was assessed using the parametric Student’s t-test and, in the case of non-normal data distribution, nonparametric tests such as the Mann–Whitney U test were employed. Categorical variables were analyzed using the χ2 test. Statistical significance was determined when the probability of the null hypothesis was less than 5% (p<0.05). Variables that marked as significant predictors for disease progression in univariate logistic analysis were subsequently subjected to multivariate binary logistic regression. To control for false discovery rate in multiple comparisons, the Benjamini–Hochberg method was applied for p-value correction. The length of survival was evaluated using the Kaplan-Meier method, while differences between groups were assessed using the log-rank test. SPSS-20 statistical software for Windows was used to calculate and process the data (Chicago, IL, USA).

## 3 Results

Study included 51 patients with a mean age of 59.83 ± 10.83 years, median 60 years (range 25–75) at enrollment. Among those enrolled, 26 (50.98%) were female and 25 (49.02%) were male, and 84.70% of the patients were in good health (ECOG performance status) (14). GEP-NETs were the most common primary tumors (52.94%), BP-NETs were being present in 21.56%, and others were unknown primary origin. There were 31 (60.78%) non-functioning NETs and 20 (39.22%) functioning NETs. Based on the Ki-67 proliferation index of the tumor, predominantly disease grade was G2 (45.10%), compared to G1 (27.45%) and G3 (27.45%). Other baseline characteristics are shown in **Table 1**. Long acting somatostatin analogues were administered to 48 (94.11%) patients.

**Table 1:** Analysis of risk factors for disease progression in patients with NETs treated with PRRT.

Variables	Progression group (n=18)	Control group (n=33)	Test and p value
Age (year)	64.10 ± 9.46	55.56 ± 12.21	Z=-1.898^***^ p=0.030; p=0.007^#^
Non-functional tumors	6	25	χ^2^= 7.892^**^ p=0.009; p=0.014^#^
Functional tumors	12	8	
Female	11	15	χ^2^= 0.759^**^ p>0.05
Male	7	18	
GEP-NET	7	20	χ^2^= 1.357^**^ p>0.05
Lung-NET	6	5	
Unknown primary origin	5	8	
NET G1	9	5	χ^2^= 1.420^**^ *p*>0.05
NET G2	7	16	
NET G3	2	12	
Hynic-TOC Krenning score <3	14	5	χ^2^= 1.820^**^ *p*=0.403
Hynic-TOC Krenning score ≥3	4	28	
Capecitabine–Temozolomide Chemotherapy before PRRT	16	8	χ^2^= 0.759^**^ p>0.05
Ki-67 (%)	19.32 ± 14.55	17.16 ± 8.90	t=0.394^*^ p>0.05
RDW (%) (red cell distribution width)	15.16 ± 1.99	14.98 ± 2.23	t=-0.196^*^ p>0.05
Erythrocytes (10^12^/L)	4.36 ± 0.55	4.35 ± 0.66	t=0.020^*^ p>0.05
WBC (10^9^/L) (white blood cell count)	6.81 ± 1,89	7.1 ± 2.75	t=0.347^*^ p>0.05
Absolute neutrophil count (10^9^/L)	5.09 ± 2.34	3.25 ± 1.20	Z=2.110^***^ p=0.044; p=0.021^#^
Absolute lymphocyte count (10^9^/L)	1.60 ± 0.81	1.98 ± 1.18	t=-0.781^*^ p>0.05
NLR (neutrophil-lymphocyte ratio)	4.66 ± 4.16	1.78 ± 1.08	t=2.397^*^ p=0.024; p=0.028^#^
Absolute monocyte count (10^9^/L)	0.73 ± 0.47	0.64 ± 0.46	t=-0.495^*^ p>0.05
Platelets (10^9^/L)	231.22 ± 66.91	228.17 ± 76.51	t=-0.111^*^ p>0.05
SIRI (systemic inflammation response index)	4.85 ± 8.38	1.52 ± 2.22	t=0.589* p =0,058
PLR (platelet-lymphocyte ratio)	226.62 ± 259.96	108.40 ± 73.93	t=0.681* p>0.05
MPV (IU/L)	9.81 ± 0.74	9.59 ± 1.61	t=-0.370^*^ p>0.05
CRP (mg/L)	11.84 ± 9.27	3.36 ± 3.14	t=2.117^*^ p=0.044; p=0.040^#^
CRP/Alb	0.34 ± 0.23	0.12 ± 0.09	t=2.607^*^ p=0.017; p=0.049^#^
Albumin (g/L)	41.36 ± 444	42.77 ± 4.30	t=0.769^*^ p>0.05

Data represent the mean value ± 1 standard deviation.

^*^Student’s t-test for independent samples.

^**^χ^2^ test.

^***^Mann–Whitney U test.

^#^Benjamini-Hochberg method of correction of unadjusted p values.

The results of our study show that the five year overall survival is 84.31%. At the time of the analysis, the median OS for both groups had not been reached, while the mean OS was 44.68 months (95% CI 37.40-51.97). Mean value of PFS was a 36.22 months (95% CI 30.14-42.29). After the introduction phase, the vast of the patients (72.47%) achieved control of the disease with SD verified in 29 (56.86%) patients and PR found in 4 (7.84%) patients, by RECIST 1.1 criteria. However, 14 (27.46%) patients had PD and death was recorded in 4 (7.84%) patients. Complete response was not observed during the five-year follow-up. The PRRT was commonly tolerated well and no grade 3 and 4 toxicity was reported, based on the National Cancer Institute Common Terminology Criteria for Adverse Events-CTCAE, version 5.0.

Univariate analysis showed that, among all variables, only 7 parameters had a statistically significant impact on the progression onset (age, functional tumors, absolute neutrophil count, NLR, CRP, CRP/Albumin, ALT). Variables that had been demonstrated the statistically significance (p<0.05) according to univariate analysis (**Table 1**), were further analyzed using multivariate logistic regression. Multivariate regression analysis emphasized that age, functionality, ALT and CRP are an independent risk factor for shorter PFS (**Table 2**).

**Table 2:** Multivariate binary logistic regression for disease progression factors in patients with NETs treated with PRRT.

Variables	Оdds ratio(95% CI)	P value
Age (year)	1.191 (0.930-1.525)	p=0.027
Functional tumors	181.56 (0,081-404833,98)	p<0.001
Absolute neutrophil count	7.993 (0.011-6076.18)	p=0.566
NLR (10^9^/L)	0.327 (0.006-16.844)	p=0.811
ALT (IU/L)	1.770 (0.445-1.334)	p=0.046
CRP (mg/L)	1.760 (0.374-1.544)	p=0.005
CRP/Albumin	46.518 (0.000-5.634)	p=0.834

**Figure 1:**
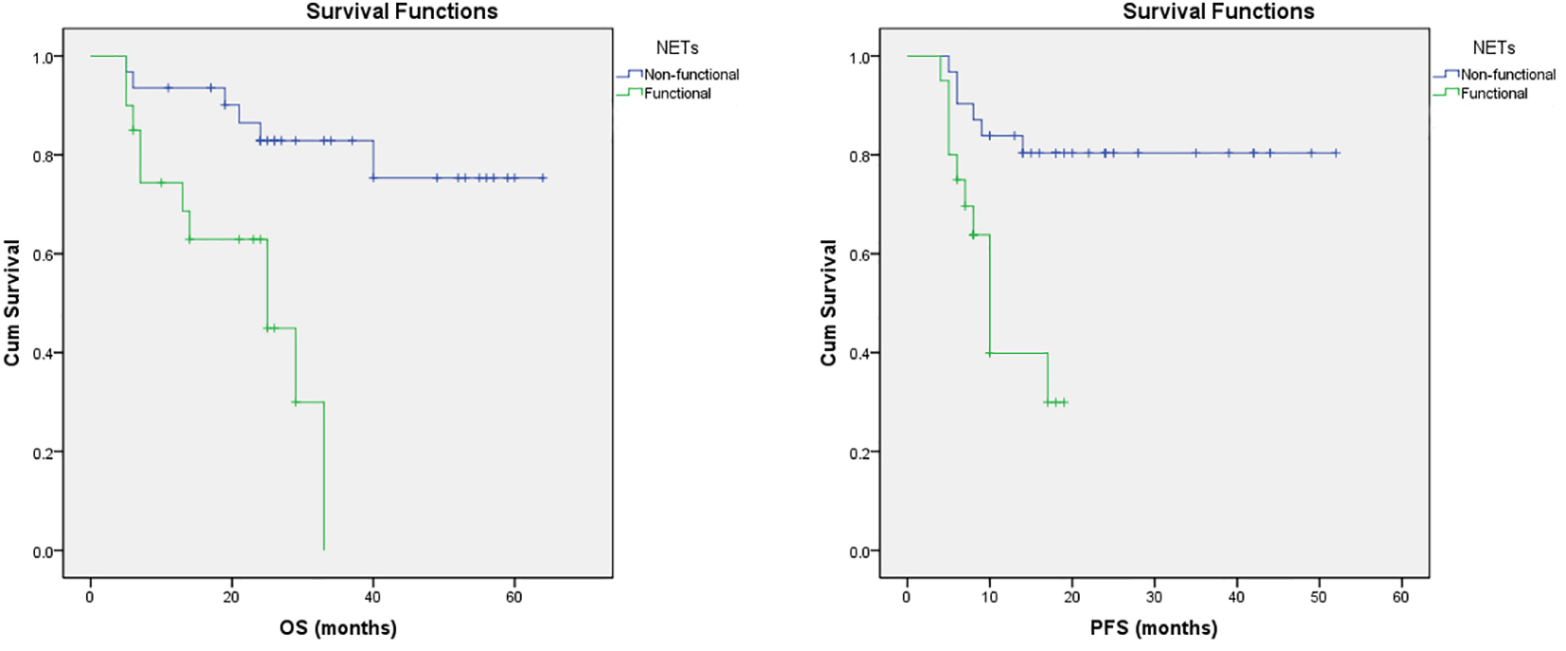
Survival trends in patients with NETs. Kaplan–Meier curves of OS and PFS in patients with NETs treated with PRRT based on their functional status.

Furthermore, multivariate regression analysis indicated that tumor functionality was the most powerful prognostic factor on the appearance of progression (p<0.001). The **Figure 1** presents that functional NETs have a lower PFS and OS. Kaplan–Meier analysis showed that for the study group, the median PFS was significantly shorter in patients with functional (11.86 ± 1.41, median 10, 95% CI: 9.06-14.64 months) than non-functional tumors (43.38 ± 3.16, 95% CI: 37.18-49.58 months), with statistical significance of p=0.001 (Mantel-Cox), HR 0.188 (0.069-0.516). In addition, OS was inversely related to tumor functionality too. In the subgroup analysis, median OS was shorter (p=0,001, Mantel-Cox) in patients with functional tumors (21.81 ± 2.70, median 25, 95% CI: 16.51-27.11 months) contrast to non-functional tumors (53.86 ± 3.70, 95% CI: 46.61-61.12 months), HR 0.155 (0.053-0.458).

## 4 Discussion

In this study, we analyzed the prediction of survival in patients treated with PRRT therapy in a cohort of 51patients with well-differentiated NETs from different sites of primary origin.

According to the literature, the median age at diagnosis of NETs typically falls within the range of 61-69 years (3, 15–19). Consistent with these findings, our study also revealed a similar pattern, with mean age of 59.83 ± 10.83. We further demonstrated that age had a statistically significant hazard ratio (1.191, 95% CI 0.930-1.525, p=0.027) affecting survival, suggesting that NETs may be more progressive in older individuals compared to younger ones. Specifically, it has been reported that patients over 40 years of age have an increased risk of death (15–17), although the clinical benefit of PRRT is satisfactory in both older and younger patients (16). In the literature, it has been shown that females tend to have better survival compared to males in the context of NETs (18, 19). However, our study did not find a significant association between gender and survival outcomes. In this study, about a half (45.10%) of NETs were G2, followed by G1 and G3 (27.45% respectively), based on the 2017 WHO criteria (12). We found that the higher the grade of the NETs were more associated with the poorer prognosis, without statistical significance between groups.

Predictive factors of PRRT response are lacking. Here, we aimed to identify predictors of treatment response by evaluating chronic inflammation markers. Chronic inflammation play an important role in the proliferation of malignant cells, angiogenesis, and metastasis of NETs and other neoplasms (8, 10, 20–26). There is increasing evidence that markers of inflammation can be used for the prognostic evaluation of various malignant tumors, including NETs. Inflammatory indexes in the blood, like NLR and PLR, are low cost, easily feasible, and can be measured repeatedly (10, 20–25). The study revealed that patients with disease progression had significantly higher levels of neutrophils, CRP, CRP/Alb and NLR. The secretion of growth factors from malignant cells causes the increased number of neutrophils in cancer patients. Additionally, neutrophils secrete cytokines that can impact the proliferation, spread, and metastasis of tumor cells (21).

Previous studies evaluated inflammatory markers like PLR, SIRI, CRP/Alb ratio, and showed that the high NLR and PLR significantly correlated with worse PFS and OS (20–25). Univariate analysis revealed that patients with an increased neutrophils count, high NLR, CRP, CRP/Alb, ALT, older patients and patients with functional NET had shorter OS and DFS. A high NLR and CRP most likely reflects an inadequate immune response that does not eliminate the tumor, but creates an environment suitable for its growth. Although the levels of these markers were higher in the study group, multivariate analysis demonstrated that only age, functionality, ALT and CRP remained significant as independent prognostic factor for disease progression and survival. ALT and AST transaminasis, reflecting the grade of liver impairment. The detected significance of ALT serum levels can be explained by reflecting liver involvement. In the literature, patients with normal ALT level had a longer PFS, suggesting that the levels of liver transaminases have a guiding effect on prognosis (26, 27).

Chromogranin A had been used as a valuable tumor marker in NETs and elevated levels of CgA and 5-HIAA as well, has previously been associated with poor prognosis was associated with poor outcome (28, 29). In the current patient population, CgA, 5-HIAA and NSE levels was not found to significantly affect survival, although higher levels of these biomarker were noticed in progression group.

The reported 5-year overall survival of 84.31% in the current study cohort was within the reported range in the literature (30–32). Mean PFS and OS in our study was 36.22 and 44.68 months respectively, which is slightly lower compared to values demonstrated in other studies (29, 32). Differences between the current study and the literature exist due to enrollment of lung and G3 NETs, treated with PRRT. The patients with GEP-NET are known to have a much better survival than patients with primary lung NETs. Other studies included only patients with G1 and/or G2 tumors, who probably have a longer OS than patients with G3 grade tumors (33).

The majority NETs are non-functional, as reported in this study (60.78%) and in the literature (60-90%) (8). Functional NETs are known to have a wide spectrum of biological and/or growth behavior. Therefore, management of functional types of NETs is very complex and remains an unmet clinical challenge. Treatment strategy often depends on the presence of various symptoms, grade of the tumor, and clinical stage (34, 35).

As shown in our series, the presence of functional NETs are associated with poor OS and PFS, respectively (**Figure 1**). Also, non-functioning tumors may alter behavior and/or become functioning and perception of this is essential concerning the strategies for the treatment options. This adds to our knowledge about PRRT in various NET groups and may help when assessing who can benefit from PRRT therapy.

In conclusion, NETs are heterogeneous group of neoplasms that could be treated with various therapeutic approach. We demonstrate that patients with well-differentiated NETs treated with PRRT, the existence of functional tumors is the major independent predictor for survival outcomes. Additionally, age, ALT, CRP, are useful independent risk factor for predicting survival in patients with NETs.

### 4.1 Limitations of study

This study had its limitations. The current series was based on a relatively small sample size, which was performed retrospectively and the heterogeneity of the patients population. However, the low incidence of NETs is well-known and the number of patients treated PRRT, so this limitation applies to many studies in the field.

## Data availability statement

The datasets presented in this study can be found in online repositories. The names of the repository/repositories and accession number(s) can be found in the article/supplementary material.

## Ethics statement

The studies involving humans were approved by Ethics Committee of the University Clinical Center Kragujevac (01/23-132). The studies were conducted in accordance with the local legislation and institutional requirements. The participants provided their written informed consent to participate in this study.

## Author contributions

VlV: Visualization, Writing – original draft, Writing – review & editing, Conceptualization, Formal analysis, Project administration, Investigation, Methodology, Supervision, Validation. KV: Investigation, Methodology, Project administration, Writing – original draft, Writing – review & editing, Data curation, Formal analysis, Validation. MZ: Data curation, Formal analysis, Software, Validation, Writing – review & editing, Methodology, Writing – original draft. ADa: Conceptualization, Data curation, Resources, Supervision, Validation, Writing – review & editing. NM: Conceptualization, Data curation, Formal analysis, Visualization, Writing – review & editing. MM: Conceptualization, Formal analysis, Supervision, Validation, Writing – review & editing. VlI: Data curation, Formal analysis, Software, Supervision, Validation, Visualization, Writing – review & editing. IS: Conceptualization, Data curation, Formal analysis, Supervision, Validation, Writing – original draft, Writing – review & editing. SD: Conceptualization, Data curation, Formal analysis, Supervision, Writing – review & editing. MS: Conceptualization, Project administration, Supervision, Visualization, Writing – review & editing. MB: Formal analysis, Investigation, Software, Validation, Visualization, Writing – review & editing. MV: Conceptualization, Validation, Writing – review & editing. ADj: Conceptualization, Supervision, Validation, Writing – review & editing. VeV: Conceptualization, Supervision, Validation, Writing – review & editing. VeI: Conceptualization, Data curation, Formal analysis, Investigation, Methodology, Validation, Visualization, Writing – original draft, Writing – review & editing.
