# Erratum: Predicting the survival probability of functional neuroendocrine tumors treated with peptide receptor radionuclide therapy: Serbian experience

**DOI:** 10.3389/fendo.2024.1369692

**Published:** 2024-01-22

**Authors:** Frontiers Production Office

**Affiliations:** Frontiers Media SA, Lausanne, Switzerland

**Keywords:** NET, PRRT, functional tumors, overall survival, progression free survival

Due to a production error, the Editor’s name and affiliation were incorrect in the published article. Instead of “Bojana Popovic, Clinic for Endocrinology, Diabetes and Metabolic Diseases, Clinical Center of Serbia, University of Belgrade, Serbia” the correct name is “Aviral Singh, GenesisCare Australia Pty Ltd, Australia”.

There was also a mistake in the Conflict of Interest statement in the originally published article. A shared affiliation between the author MB and the wrong Editor was erroneously disclosed. The correct statement appears below.

“The authors declare that the research was conducted in the absence of any commercial or financial relationships that could be construed as a potential conflict of interest.”

The publisher apologizes for this mistake. The original version of this article has been updated.

